# The role of the retinoids in schizophrenia: genomic and clinical perspectives

**DOI:** 10.1038/s41380-019-0566-2

**Published:** 2019-10-30

**Authors:** William R. Reay, Murray J. Cairns

**Affiliations:** 1 School of Biomedical Sciences and Pharmacy, The University of Newcastle, Callaghan, NSW Australia; 2 Centre for Brain and Mental Health Research, Hunter Medical Research Institute, Newcastle, NSW Australia

**Keywords:** Molecular biology, Schizophrenia, Neuroscience, Genetics

## Abstract

Signalling by retinoid compounds is vital for embryonic development, with particular importance for neurogenesis in the human brain. Retinoids, metabolites of vitamin A, exert influence over the expression of thousands of transcripts genome wide, and thus, act as master regulators of many important biological processes. A significant body of evidence in the literature now supports dysregulation of the retinoid system as being involved in the aetiology of schizophrenia. This includes mechanistic insights from large-scale genomic, transcriptomic and, proteomic studies, which implicate disruption of disparate aspects of retinoid biology such as transport, metabolism, and signalling. As a result, retinoids may present a valuable clinical opportunity in schizophrenia via novel pharmacotherapies and dietary intervention. Further work, however, is required to expand on the largely observational data collected thus far and confirm causality. This review will highlight the fundamentals of retinoid biology and examine the evidence for retinoid dysregulation in schizophrenia.

## Introduction

Schizophrenia is a psychiatric disorder likely influenced by an array of genetic and environmental factors [1–4]. However, clinical management remains challenging and little progress has been made in identifying treatment targets beyond the traditional anti-psychotic paradigm. Development of novel treatments is confounded by the complex aetiology of schizophrenia; wherein multiple biological systems are involved, and their relative contribution to pathophysiology may be specific to the individual, their genetic makeup, and/or specific exposures to biological or psychosocial stress. This is consistent with the long-standing neurodevelopmental hypothesis of schizophrenia, whereby an interplay between genetic and environmental factors disturb early neurodevelopment [5–7]. This seems to correspond to a reduction of dendritic volume and density, which manifests macroscopically as cortical thinning and reduced neural connectivity [8–10]. While early investigation of this hypothesis was focused around the dopamine system, because of its clear role in psychosis, it now seems likely that there are many biological pathways involved in schizophrenia and related psychotic disorders. Therefore, to address this, we need to adopt a systems approach that can deconvolve the disorder into these biological pathways, particularly those that can be manipulated by existing drugs or are accessible targets for drug development. There is one system, in particular, involved in retinoid biosynthesis and signalling, that is a strong contender for consideration in the context of schizophrenia because of its well-known role in neural development via control of neuronal differentiation. Retinoids are metabolites of vitamin A (all-*trans* retinol)—commonly referred to as retinol, a fat soluble vitamin derived from foods in active form or as provitamin precursors such as a beta-carotene [11]. All-*trans* retinoic acid (at-RA) is the most biologically active retinoid and exerts a number of vital genomic and non-genomic effects [12, 13]. Retinoids play a role in a suite of developmental pathways but also are significant for a number of post-natal processes including synaptic plasticity [14, 15], lymphocyte homoeostasis [16, 17], inflammation [18], and the visual cycle [19] as only a few such examples. Interestingly, they can also be modulated by both natural and synthetic agonists and antagonists, making it a putative treatment target. In this review we discuss the retinoid system and its dysregulation in the context of schizophrenia. We also consider the development of biomarkers for retinoid system dysfunction and their application in guiding precision application of dietary and pharmacological interventions for schizophrenia.

## Systemic transport of retinoids

**Fig. 1:**
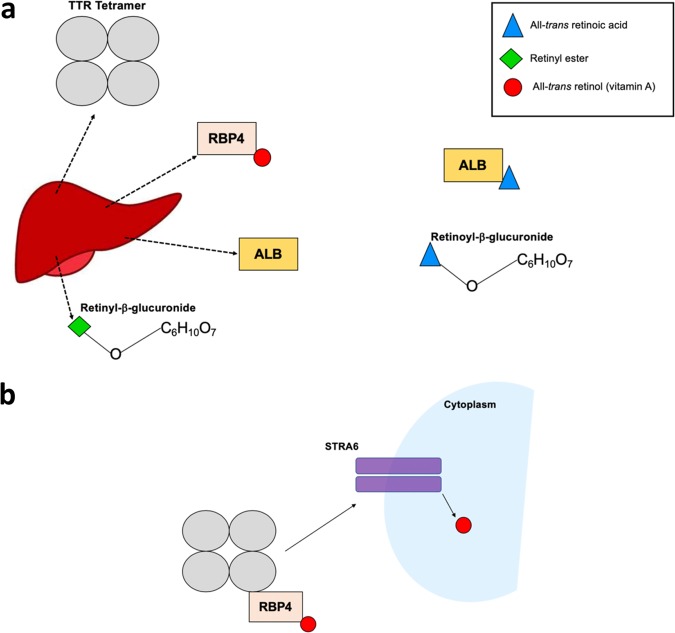
Schematic of mechanisms for systemic retinoid transport. **a** Vitamin A (all-*trans* retinol) is secreted by hepatocytes bound to retinol binding protein 4 (RBP4). Hepatic retinol can also be esterified (retinyl ester) and transported linked to glucuronic acid (C_6_H_10_O_7_) via a glycosidic bond – forming a retinyl-β-glucuronide. Further, all-*trans* retinoic acid is also found in serum, including bound to albumin (ALB) protein of hepatic origin and as a β-glucuronide complex. **b** Transthyretin (TTR) complexes with RBP4 bound retinol for systemic transport. The surface receptor protein stimulated by retinoic acid 6 (STRA6) is postulated to interact with the TTR:RBP4:Retinol complex, facilitating intracellular influx of retinol

Vitamin A is packaged primarily for serum transport in the liver where the major storage form is as esterified retinol (retinyl esters) within hepatic stellate cell lipid droplets [20, 21]. Retinol binding protein 4 (RBP4) is the prinicipal retinol serum transporter with ~95% of all retinol shown to be trafficked in this manner [22] (Fig. 1a). Hepatocytes secrete retinol bound to RBP4, which in turn complexes in serum with the tetrameric protein transthyretin (TTR). TTR, once complexed with RBP4 bound retinol, plays an important role in determining serum retinol concentration via downregulating renal filtration of the RBP4 protein [23]. While the mechanisms underpinning cellular retinol influx from the TTR/retinol/RBP4 pathway are not fully resolved, the membrane protein STRA6 (stimulated by retinoic acid 6) has been implicated in bi-directional retinol transport across the cell membrane [24, 25], with decreased retinol concentrations observed in homozygous *Stra6*^*−/−*^ knock-out mice in several tissues including the brain and retina [26, 27] (Fig. 1b).

Whilst the RBP4 holoenzyme is integral to the transport pathway, several auxiliary mechanisms exist. Retinyl esters, as opposed to active retinol, can be packaged for serum transport within lipoproteins or conjugated to glucuronic acid via a glycosidic bond, forming retinyl-β-glucuronides [28, 29]. At-RA is also detected at low serum concentrations either bound to albumin or as a retinoyl-β-glucuronide complex [30]. Regarding transport to the brain, the role of these secondary pathways is still not well characterised. STRA6 is highly expressed by the blood-brain barrier (BBB), which suggests the RBP4-TTR mechanism facilitates entry into neural tissues—supported by in vivo evidence from *Stra6*^*−/−*^ mice where neural retinol homoeostasis is disturbed [24, 26, 27]. As retinol delivery to the brain is essential for subsequent at-RA biosynthesis, specific factors which may regulate transport and passage across the BBB should be investigated further, particularly as localised neural retinoid deficiency would be of particular phenotypic relevance to schizophrenia.

## Enzymatic biosynthesis of retinoic acid

**Fig. 2:**
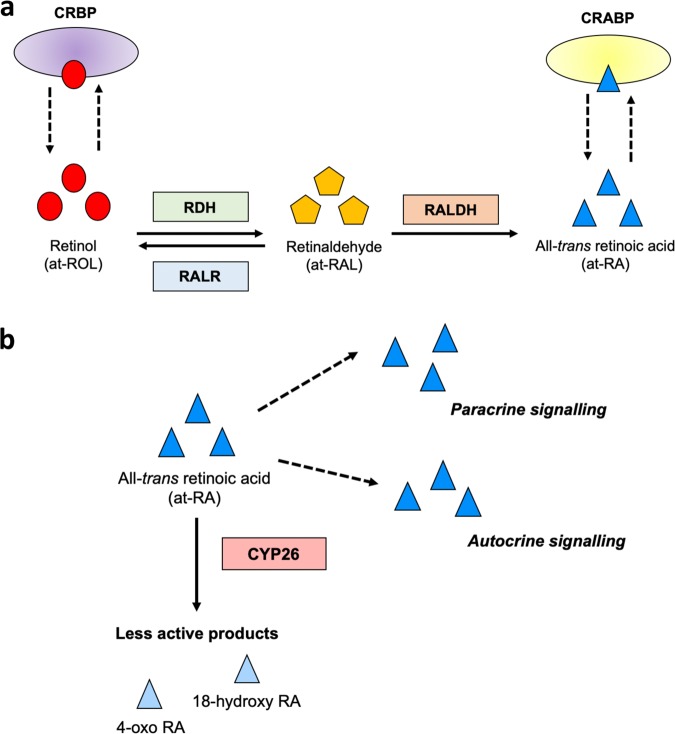
Biosynthesis and clearance pathway of all-*trans* retinoic acid (at-RA). **a** Retinol (vitamin A) complexes with cellular retinol binding proteins (CRBP) for intracellular transport. The first stage of at-RA synthesis is the conversion of retinol to retinaldehyde via the oxidative capacity of retinol dehydrogenase (RDH) family enzymes. Retinaldehyde reductases (RALR) can reverse this process as a rate limiting step in at-RA production and reduce retinaldehyde back to retinol. Finally, retinaldehyde is oxidised to at-RA upon the activity of retinaldehyde dehydrogenases (RALDH) and binds to its own cellular transport protein (CRABP). **b** Once synthesised at-RA may signal in an autocrine or paracrine manner. CYP26 family enzymes can deactivate at-RA by catabolism into less bioactive compounds including 4-oxo retinoic acid and 18-hydroxy retinoic acid. This acts as a regulator of at-RA abundance and is important for maintenance of cellular homoeostasis

The pathway which produces at-RA from its retinol precursor encompasses a series of redox reactions catalysed by distinct enzyme families (Fig. 2a). Once imported into the cell, cellular retinoid binding proteins (CRBP) bind retinol to protect from non-specific oxidation and, therefore, providing the substrate for subsequent at-RA synthesis [31]. Initially, retinol is oxidised to an intermediate form, all-*trans* retinal (at-RAL)—commonly referred to as a retinaldehyde, via the action of cytosolic retinol dehydrogenase enzymes such as RDH12 [32, 33]. Retinaldehyde synthesis is reversible and is an important rate limiting step in at-RA production [34]. Various short-chain and aldo-keto reductases, broadly classified as retinaldehyde reductases (RALR), are able to catalyse this reduction of retinaldehyde back to retinol [35].

Retinaldehyde is thereafter oxidised to form at-RA through the action of retinaldehyde dehydrogenases (RALDH): specifically, ALDH1A1, ALDH1A2, and ALDH1A3 have been shown to be the functional RALDH enzymes in vivo [33]. Analogous to CRBP, synthesised at-RA complexes with its own binding protein (cellular retinoic acid binding protein [CRABP]) and exerts its biological functionality in an autocrine or paracrine manner [36]. Due to the biological potency of at-RA, catabolism into less active isoforms are essential to maintain cellular homoeostasis. Cytochrome P450 family 26 enzymes (CYP26) facilitate this by converting at-RA into products including 18-hydroxy retinoic acid and 4-oxo retinoic acid [37] (Fig. 2b). Synthesis and clearance of at-RA remains tightly controlled as both deficient and excess concentrations can be pathogenic. Indeed, at-RA influences the expression of several enzymes involved in these catabolic processes, forming a feedback loop which can therefore modulate its cellular abundance [38]. The regulatory control on transcription induced by at-RA underlies this feedback mechanisms and involves discrete receptor signalling pathways once at-RA is bound to a receptor.

## Signalling by retinoic acid regulates gene expression

**Fig. 3:**
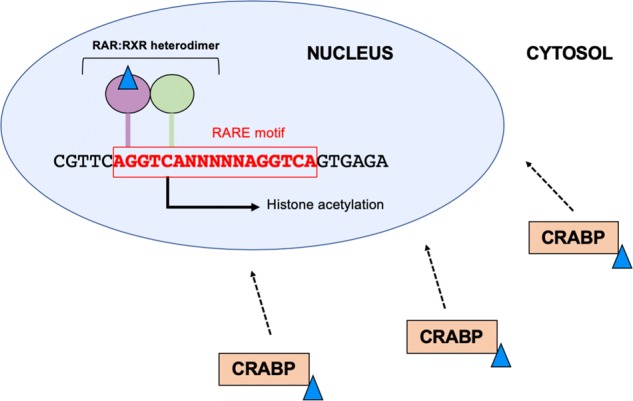
Receptor signalling pathway for all-*trans* retinoic acid (at-RA). Cellular retinoic acid binding proteins (CRABP) protect at-RA from non-specific degradation and transport it to the nucleus. Retinoid receptors recognise and bind to a DNA sequence motif termed a retinoic acid response element (RARE). This motif is composed of two repeats of a hexameric motif, separated by a variable number of nucleotides (N)—in this schematic we show a five-nucleotide separator sequence (DR5-RARE) as highlighted in red. Once at-RA is liganded to a receptor, this initiates a signalling cascade in the canonical mechanism whereby nuclear co-activators are recruited. Expression of proximal genes to the RARE sequence is upregulated via the action of the co-activators which alter chromatin availability, for example, histone acetylation

Three classes of nuclear receptors exist for which retinoic acid acts as a ligand: retinoic acid receptors (RAR), retinoid X receptors (RXR), and the peroxisome proliferator-activated receptor beta/delta (PPARβ/δ) [39–41]. RAR and RXR further both have three distinct subtypes (alpha, beta and gamma), and therefore seven established retinoid receptors have been characterised in total. These receptors bind to the DNA itself via distinct sequence motifs termed retinoic acid response elements (RARE). RARE sequences are comprised of two direct repeats of a hexameric motif which may be separated by a variable number of nucleotides—one of the most well characterised RARE being the motif pair separated by five variable nucleotides (DR5-RARE) [42]. Ligand binding elicits a conformational change which initiates at-RA signalling, specifically, co-activators including histone acetyl transferases are recruited which promote transcription of proximal genes to the DR5-RARE/receptor complex [12, 43] (Fig. 3). RAR subtypes exist bound to RAREs as a homodimer or they alternatively heterodimerise with RXRs; however, RXRs have a variety of other binding partners including the vitamin D receptor (VDR), thyroid receptor (TR) and several PPAR isoforms [40]. This reinforces that components of the retinoid signalling apparatus may influence the targets of many other DNA binding proteins, and, therefore, a wider variety of genes than those with a functional RARE.

The precise number of genes influenced by at-RA across the breadth of tissues and cell-types in which it functions is still not precisely quantified, although genome-wide chromatin immunoprecipitation sequencing (ChIP-seq) has elucidated that binding peaks for receptor subtypes exist in over ten thousand genes [44, 45]. ChIP-seq output is, however, cell type specific and several retinoid receptors, along with their various dimers, have yet to be profiled. In silico prediction of the positions of DR5-RARE by Lalevée et al. further supports a multitude of biological retinoid targets as over 15,000 genome-wide putative DR5-RARE were identified, of which around 3000 were within 10 kilobases of a coding exon [42]. As thousands of genes are predicted to have their transcription impacted by at-RA, dysregulation of this pathway would likely have widespread consequences depending on the temporal and tissue specific nature of the alteration.

## Retinoic acid in the human brain

Retinoic acid signalling has important implications for normal neuronal function throughout the human lifespan. Concentration of at-RA is tightly regulated depending on homoeostatic feedback from the cellular environment, with even slight deviations potentially triggering pathological consequences. This is particularly critical as either apoptotic or pro-survival cascades can be induced by at-RA dependent on which receptor subset is liganded [46]. In utero at-RA acts as a potent morphogen, with concentrations of 0.5 μM shown as sufficient to induce neuronal differentiation from embryonic stem cells (ESCs) [12, 47, 48]. Dynamic concentration gradients during embryogenesis thus influence the neuronal fate of ESC progenitors as well as specification of position. For instance, Okada et al. demonstrated dorsal and ventral like phenotypes arise from higher and lower at-RA concentrations respectively [49]. As neuronal structures mature, there is a corresponding increase in neurovascular complexity to supply nutrients necessary in order to sustain metabolic demand. At-RA also acts as an important regulator of cerebrovascular development, which consolidates the essentiality of embryonic retinoid homoeostasis [50].

Embryonic actions of at-RA have long been characterised, however, its significance within the adult brain has only more recently been established. Whilst disruption of neuronal development has clear linkage with psychiatric illness, at-RA remains clinically relevant when considering its function throughout the lifespan. This includes its involvement in synaptic plasticity—an example of which being the process of homoeostatic synaptic plasticity (HSP) [51]. HSP is employed by neural circuits to stabilize activity in response to increased modulation. In periods of prolonged inhibition, decreased calcium concentration blocks the action of the phosphatase calcineurin—in turn directly upregulating at-RA synthesis. Interestingly, HSP involves a non-genomic method of at-RA signalling, as once liganded, the retinoid receptor complex influences the synthesis of synaptic proteins by directly binding to mRNA [15, 52]. Murine studies have also demonstrated that calcineurin knock-out is associated with schizophrenia associated phenotypes including abnormal behaviour and impaired working memory [53, 54]. A deficit in at-RA synthesis or neuronal availability of retinoid precursors may therefore dysregulate HSP, however, this is yet to be investigated in schizophrenia and other neuropsychiatric disorders. Interestingly, at-RA directly regulates expression of enzymes within the dopamine synthetic and metabolic pathways, including tyrosine hydroxylase and dopamine-β-hydroxylase [55]. The dopaminergic receptor *DRD2*, the principal anti-psychotic target for schizophrenia, is also proximal to a RARE motif and predicted to be regulated by at-RA [56].

Several cognitive domains have been extensively linked with retinoid function. Murine retinoid receptor knock-out models demonstrate impaired or absent hippocampal long term potentiation and depression, the effect size of which being somewhat dependent on the receptor subtype ablated [14]. Further evidence supports that retinoids are intrinsic to the maintenance of cognitive performance. Post-natal vitamin A deficiency is associated with impaired relational and spatial memory [57, 58]—a deficit which in rodents can be reversed by retinoid supplementation [59, 60]. This may be significant in schizophrenia, as cognitive deficits respond poorly to conventional antipsychotic pharmacotherapy and the retinoid system may act as a modifiable enhancer of cognitive performance.

## Retinoic acid in the regulation of inflammation and immunological tolerance

Inflammatory processes are now recognised as an important risk factor in the aetiology of schizophrenia [61–64]. As retinoid signalling plays a significant role in immune cell function it is plausible that factors that affect this system could have important implications for schizophrenia-associated inflammatory stress. For instance, signalling by at-RA is integral to the differentiation of a regulatory specialised population of T lymphocytes (T_reg_) [65, 66]. This T_reg_ subpopulation exerts an immunosuppressive effect via downregulation of effector T lymphocytes and, thus, prevents aberrant inflammation and autoimmunity [67]. Retinoids further act as regulators of the blood-gut barrier—a vital structure for preventing inflammation in response to the gut microbiome [68]. Aberrant permeability (‘leakiness’) in this barrier has been linked with several inflammatory conditions and may also have implications for adverse immune responses associated with schizophrenia [69, 70]. A more direct link between retinoids and neuroinflammation is mediated by the action of at-RA on astrocyte and microglial populations, whereby at-RA downregulates their activity [71]. As a result, retinoid perturbations have been linked with several autoimmune disorders such as multiple sclerosis [72].

## Biochemical and clinical evidence of retinoic acid dysregulation schizophrenia

A substantial body of evidence has accumulated to suggest retinoids are somehow dysfunctional in schizophrenia. Both distinct biological factors and clinical outcomes have been characterised to support this hypothesis. Seminal research by Ann B. Goodman in the 1990s proposed three central lines of evidence as indicative of at-RA involvement in schizophrenia pathogenesis [73]: (i) neurological congenital abnormalities reported in some schizophrenia cases are comparable to those observed with vitamin A deficiency or altered at-RA [74], (ii) genetic linkage studies have implicated retinoid associated loci in the genome [75], and (iii) proposed schizophrenia risk genes contain a putatively functional RARE motif [75]. The precise genes studied by Goodman with linkage-based approaches have not been validated with the advent of large-scale molecular studies of schizophrenia cohorts, however, several other retinoid genes have now been shown to be rigorously associated with the disorder. More recent epidemiological data provide some preliminary evidence of deficits in maternal retinol increasing the risk for the development of schizophrenia in offspring [76]. This study tested the association between maternal retinol levels in the second and third trimester and their offspring developing schizophrenia, with serum samples sourced from prenatal determinants of schizophrenia study cohort [77]. Samples from mothers whose offspring developed a schizophrenia spectrum disorder (*n* = 55) were analysed relative and matched maternal controls (*n* = 106, up to two matched controls per case). Interestingly, adjusted for maternal age and education, this effect was only statistically significant for maternal retinol concentration during the second trimester, which the developing rodent brain, in an analogous pre-natal time period, demonstrated particular sensitivity to retinoid perturbations [78]. To our knowledge, further study of this area has yet to be published, and replication in a larger cohort is required to provide support to these findings. In addition, the observational nature of this study means that no definitive causal link between maternal retinol levels and schizophrenia can be currently established.

## Genomic characterisation of retinoid dysregulation in schizophrenia

The genomic architecture of schizophrenia is multifactorial (polygenic), with contributions from many variants genome wide, the frequency of which ranging from common to ultra- rare in the population [4, 79]. Initial genomic studies of schizophrenia were hampered by small sample sizes and relied on linkage approaches to identify candidate genes.

High throughput common variant genotyping has enabled the construction of large-scale genome-wide association studies (GWAS) by collaborative consortia such as the Psychiatric Genomics Consortium (PGC). A GWAS is usually a univariable test of association between a genomic marker (single nucleotide polymorphism [SNP]) and a phenotype, in this instance schizophrenia. Utilising this approach has revealed almost 150 common loci as significantly associated with schizophrenia after rigorous multiple testing correction (*P* < 5 × 10^−8^ as the significance threshold) [3, 80]. Notably, five of the significant variants identified span a locus which contains a gene involved in retinoid biology (Table 1). Whilst other systems important in schizophrenia pathophysiology, such as glutamatergic and dopaminergic signalling are also implicated by the GWAS, it is logical that there are many salient pathways in a disorder like schizophrenia with complex pathophysiology. The most significantly associated of these SNPs is found within the gene *RERE* (arginine-glutamic acid dipeptide repeats)—which encodes an atrophin family protein that acts as an important in utero regulator of at-RA [81]. The marker SNP for this locus was also subsequently nominally associated with an attentional control cognitive task in a schizophrenia cohort [82]. In addition, this gene has also been implicated by GWAS in neuroticism and depressive symptoms [83], whilst the deletion of its chromosomal locus 1p36 causes a congenital disorder characterised by symptoms including intellectual disability (ID) [84]. The next two most significant retinoid schizophrenia SNPs are physically mapped to the *ZNF536* and *CYP26B1* genes respectively. *ZNF536* is a zinc finger nuclease with particular abundance in the developing brain which regulates at-RA during neuronal differentiation [85]. Whilst this zinc finger nuclease can target many genes, it’s central function thus far is attributed to retinoid biology. Degradation of at-RA into less bioactive compounds is also important for the maintenance of cellular homoeostasis—*CYP26B1* being one such enzyme involved in this degradation process [37]. Interestingly, at-RA catabolism by *CYP26B1* is also specifically implicated as important for T-lymphocyte localisation and trafficking [86]. A further regulatory component, the CCR4-NOT transcription complex subunit 1 (*CNOT1*), which can supress RXR mediated signalling, also contains a genome-wide significant schizophrenia variant [87]. Overall, single common variants in the retinoid pathway seem to map to disparate components of regulatory mechanisms acting on retinoids. This is notable as slight perturbations in retinoid availability conferred by these disruptions in its regulatory architecture would likely be deleterious.

**Table 1:** Marker SNPs associated with schizophrenia (from the psychiatric genomics consortium mega-GWAS) within a haplotype to which a retinoid related gene was mapped

SNP ID	Annotation	Retinoid gene
rs34269918	Intron variant	*RERE*
rs2053079	Intron variant	*ZNF536*
rs3768644	Intron variant	*CYP26B1*
rs8082590	Intron variant	*RAI1*^a^
rs12325245	Intergenic variant	*CNOT1*

^a^Marker SNP (rs8082590) located within *GID4* gene but implicated haplotype features the retinoic acid induced 1 (*RAI1*) locus. Adapted from Reay et al. [96]

It should be noted that GWAS loci can be difficult to readily interpret: a GWAS association traditionally is discovered with either a variant physically genotyped or imputed from a SNP microarray (marker SNP). However, this marker SNP actually represents a cluster of co-inherited variants in the genome called a haplotype. This becomes even more complex biologically in multigenic GWAS loci, in which these co-inherited SNPs span multiple proximally located genes. As a result, the schizophrenia marker SNP rs8082590 is potentially relevant to the retinoid pathway. This variant is mapped to an undifferentiated locus encompassing several genes including *RAI1* (retinoic acid induced 1), and thus, functionally significant variation may impact this gene. Expression of *RAI1* is mediated by retinoic acid and is suspected to act as a transcriptional regulator, although its precise function remains poorly characterised [88]. Highly expressed in neurons, it presents as an interesting functional candidate as it is a probable causal gene for two disorders which are characterised by phenotypes relevant to schizophrenia including ID and behavioural abnormalities. These disorders are Smith Magenis Syndrome and Potocki-Lupski Syndrome, attributed to haploinsufficiency and duplications of *RAI1* respectively [89, 90]. Further support to the neuropsychiatric relevance of this gene is also evidenced by its implication in non-syndromic autism spectrum disorders (ASD) [91–93].

The polygenic nature of complex disorders like schizophrenia means that variants which do not exceed genome-wide significance in GWAS (*P* > 5 × 10^−8^) are likely also important to its neurobiology. Univariate effect sizes of even strongly associated common SNPs are small, however, the combined effect of many SNPs which do not individually survive genome-wide correction may be more clinically meaningful [79]. This is notable as it implicates multiple components of retinoid biology at a network level. Leveraging this concept can provide important insights into the enrichment of variation within defined genes and biological pathways. For instance, gene-based tests, as opposed to single marker GWAS, investigate the joint impact of all independent SNPs mapped to a gene [94, 95]. The five retinoid genes mapped to GWAS loci as described above were also associated with schizophrenia using a multivariable gene-based test [96]. Common variant enrichment within several gene ontology sets related to vitamin A was also observed in a Danish schizophrenia cohort utilising another multi-marker approach [97]. We further demonstrated that a polygenic risk score constructed from retinoid gene variation not exceeding genome-wide significance was associated with the disorder, suggesting that there is evidence of a larger polygenic signal in this system [96]. All retinoid related GWAS loci require further investigation, both to characterise functional SNPs and elucidate how retinoid biology may be impacted. However, polygenicity, comprising many SNPs within the retinoid pathway, is likely particularly important as this may encompass alterations in several retinoid related processes and capture inter-individual genomic heterogeneity.

Variation which occurs infrequently in the population (rare variants), are predicted to have a much greater individual effect size than common loci [98]. Exome sequencing of schizophrenia probands and their unaffected parents has uncovered de novo rare variation (spontaneously occurring in the proband) mapped to retinoid genes which could be clinically significant for that individual [99]. Genes enriched for predicted deleterious de novo rare variation in schizophrenia were also overrepresented for genes which interact with at-RA [100]. Whole genome sequencing (WGS) provides an opportunity to more accurately assess the impact of rare variants in schizophrenia. Non-coding regions of the genome with important regulatory functions are available in WGS output and this includes the RARE motifs which mediate at-RA signalling. Preliminary analyses using WGS in schizophrenia have demonstrated: (i) evidence of genome-wide enrichment of rare variation altering the sequence of DR5-RARE, and (ii) an association of rare variation in the retinoic acid receptor beta (*RARB*) gene with a severe cognitive deficit subtype of the disorder [96]. Larger sample sizes are required to replicate these findings but taken together these analyses suggest a role for common and rare variation impacting retinoid genes in schizophrenia pathogenesis. The heterogeneous nature of rare variation likely indicates that the functional consequences will be highly patient specific. For instance, variation which blocks receptor binding to the DR5-RARE sequence will have a different effect dependent on proximal genes regulated by at-RA and the local cellular microenvironment. Future research is required to further examine how these different variant types act on retinoid biology and whether this can be leveraged for precision interventions for individuals.

## Differential expression of retinoid related transcripts in schizophrenia

Analyses of gene expression has provided further evidence to support a role for retinoids in schizophrenia pathophysiology. Early work using gene expression microarrays to quantify transcriptomic alterations identified several retinoid genes differentially expressed in post-mortem neural tissue. For instance, in a 2005 study albumin (*ALB*) and the RALDH gene *ALDH1A1*, which oxidises retinaldehyde to at-RA, were significantly downregulated in schizophrenia [101]. Next generation sequencing of RNA (RNAseq) has now been applied to much larger cohorts, with better power to detect dysregulated transcripts in the disorder. Recent sequencing of post-mortem cerebral cortex samples, encompassing over 500 schizophrenia cases, revealed differential expression of several genes integral to the retinoid pathway after transcriptome wide multiple testing correction [102]. These include genes with corresponding genomic evidence, including downregulation of *CYP26B1* and *RAI1* and upregulation of *RERE*. Thus, further support is provided to a perturbation of regulatory processes influencing retinoids in schizophrenia as at-RA catabolism may be decreased via impaired *CYP26B1*, with a potential influence on the rate of at-RA signalling by *RERE*. Downregulation of *ALDH1A1* was also replicated in this study along with decreased transcription of *RDH11*, suggesting a disruption to at-RA synthesis itself. The retinaldehyde reductase *DHRS3* was additionally upregulated and, therefore, may also negatively impact neuronal at-RA availability. Interestingly, weighted gene correlation analysis in this study also consolidated the involvement of retinoid biology, with co-expression modules associated with schizophrenia significantly enriched for retinoid processes after correction including retinol transporter activity and negative regulation of at-RA signalling.

Systemic dysregulation of gene expression in schizophrenia is also considered to be an important aspect of the disorder—particularly, as systemic abnormalities including aberrant inflammation have now been implicated [61–63]. Sequencing of a large sample of patient derived lymphoblastoid cell lines supported systemic dysregulation of retinoid biology [103]. Fused in Sarcoma (*FUS*) has been shown to act as a negative repressor of at-RA signalling via the RAR:RXR heterodimer during haematopoiesis and had significantly increased expression in schizophrenia samples [103, 104]. Two retinoid receptors, *RARA* and *RXRA*, were also upregulated systemically, which could possibly be a homoeostatic response to an overall phenotype of retinoid perturbations [103, 105]. Another of Goodman’s core retinoid hypotheses has further been supported by RNA sequencing, with several differentially expressed transcripts in schizophrenia that are proximal to a predicted DR5-RARE motif [73, 96]. It should be noted that as RARE are predicted to regulate thousands of transcripts, a putative risk gene for schizophrenia located proximal to a RARE does not directly provide evidence of a role for retinoids in schizophrenia. However, genes regulated by RARE may provide mechanisms whereby retinoid dysregulation is involved in schizophrenia pathogenesis. For instance, differentially expressed genes in schizophrenia proximal to a RARE have been shown to be overrepresented in pathways related to neurodevelopment and inflammation, which are likely important for the disorder [96].

## Clinical interventions in schizophrenia targeting the retinoid system

The summation of these data from the literature suggest potential utility of retinoid-based therapies in clinical practice. Firstly, retinoids are a particularly attractive treatment target as this system can be modulated by dietary intervention. Foods rich in pre-formed vitamin A include liver, oily fish and some cheese-products—whilst many vegetables are a good source of beta-carotene, which can be converted into retinol. Interestingly, intake of beta-carotene was demonstrated to be low in some schizophrenia cohorts [106]. Direct pharmacological activation of retinoid receptors may, however, present as more powerful approach to rescue retinoid homoeostasis.

Data from two clinical trials has suggested that a synthetic RXR specific agonist, Bexarotene, may be an affective adjuvant for schizophrenia [107, 108]. This compound acts via the pro-apoptotic RXR mechanism, however, low doses of the treatment were well tolerated, with significant improvements observed in those prescribed bexarotene using the Positive and Negative Symptom Scale (PANSS) score as a primary outcome. However, these results require replication in a larger trial to confirm the efficacy and safety of this intervention. A precision approach leveraging genomics would likely greatly enhance the implementation of novel pharmacotherapies like bexarotene in schizophrenia. Heterogeneous drug response has confounded many interventions and those at heightened genomic risk for retinoid dysregulation may disproportionately benefit from a synthetic retinoid and/or dietary approach. For example, patients with elevated polygenic common variant risk within retinoid genes may have increased liability for altered retinoid homoeostasis. Genotypes of the genes which encode RXRs (*RXRA*, *RXRB*, and *RXRG*) should also be considered, as disruptive variation which impairs receptor binding may preclude patients from utilising bexarotene—saving resources and unnecessary side-effects as a result. Other retinoid compounds in addition to bexarotene may also warrant investigation. For instance, previous drug orientated enrichment of protein–protein interaction networks between cortical differentially expressed genes in schizophrenia suggests that compounds which act via RARs, such as tamibarotene, may also be candidates [109]. Larger trials of retinoid compounds in schizophrenia are needed to confirm their efficacy and integration of genomics into these trials will likely be important for resolving true clinical utility.

## Retinoids in other psychiatric disorders

High genetic correlation has been uncovered between different neuropsychiatric traits, which may account for some of the observed overlaps between clinical presentation [110]. Therefore, it is not surprising that retinoids have also been implicated in other psychiatric disorders and neurological traits. As retinoids play an intrinsic role in embryonic development, several developmental disorders characterised by symptoms relevant to schizophrenia, including ID, have been attributed to retinoid related variation [84, 89, 90, 111]. In ASD, preliminary evidence suggests that serum retinol is lower in affected individuals, although it is unclear whether this arises from altered diet or deficits in retinol metabolism [112]. Copy number variation of the 15q11-13 region, a well characterised risk factor for ASD, is also postulated to perturb retinoid homoeostasis via downregulation of *ALDH1A2* conferred by duplication of the E3 ubiquitin ligase gene *UBE3A* [113, 114]. The role of retinoids in affective disorders remains controversial, particularly as the synthetic at-RA stereoisomer 13-*cis* retinoic acid [isotretinoin], indicated for dermatological conditions, has been associated with suicidality [115]. In vivo evidence supports this assertion [116, 117], particularly as isotretinoin has opposing effects to at-RA on important neurological processes including hippocampal neurogenesis [118]. However, recent meta-analysis did not reveal a statistically significant association between isotretinoin use and the risk of depressive disorders (RR = 1.15, 95% CI 0.60–2.21, *P* *=* 0.14), although this effect was discordant between prospective (RR = 0.85, 95% CI 0.60–2.21, *P* *=* 0.86) and retrospective (RR = 1.39, 95% CI 1.05–1.84, *P* *=* 0.02) studies individually [119]. Retinoid related pathophysiology specific to schizophrenia remains difficult to define, particularly due to the heterogeneity of clinical presentation within the disorder. It should be noted that for psychiatric disorders subjected to GWAS, schizophrenia thus far displays the most extensive common variant association with retinoid biology. Particularly as there appears to be a polygenic signal affecting this system beyond genome-wide significant SNPs [96]. Future research is required to more precisely dissect downstream consequences of retinoid dysfunction which may be characteristic of schizophrenia. Intuitive candidates include dopaminergic signalling, for which retinoids play an established regulatory role [56, 120].

## Future directions

There is likely to be continued interest in the retinoid system in psychiatry, particularly as retinoids act as master regulators of many important processes both neurologically and systemically. We have outlined in this review multiple lines of evidence to support a role of retinoid biology as a component of schizophrenia pathogenesis, however, more work is required to elucidate the underlying mechanisms and leverage this for treatment. This is particularly true because much of the data described in this review arises from observational studies, and thus, may be biased by confounding effects. Firstly, whilst there is significant evidence of genetic perturbations in the retinoid system being associated with schizophrenia, molecular analyses are now required to investigate the functionality of this variation. For example, newly emergent genome editing techniques such as CRISPR-Cas9 present an opportunity to model risk variation in retinoid genes via the generation of isogenic cells lines or in vivo animal models. We believe also that more epidemiological data needs to be collected to accurately assess the relationship between serum retinol concentration and schizophrenia. Previous work using dried neonatal blood spots has demonstrated that neonatal vitamin D deficiency was associated with later-life schizophrenia [121]. This approach could also be applied in this context to large cohorts for which blood spots are available, as retinol can be accurately assayed from dried blood [122, 123]. Finally, it is essential that more data is collected to quantify whether there is a causal relationship between retinoid dysregulation and schizophrenia. Whilst the results of initial clinical trials of bexarotene have been promising, the relatively small sample size of these studies necessitates replication in larger cohorts. As a result, these initial data should be treated as preliminary. Due to the immense inter-patient heterogeneity, trials ideally should adopt a precision approach in the allocation of retinoid pharmaceuticals, as those with elevated genic risk affecting the retinoid pathway will likely benefit most readily from this approach. Retinoid analogues which act through RAR rather than RXR, such as tamibarotene, may also warrant investigation for use as an adjuvant in the disorder. Genetic instrument variables for vitamin A levels could further be utilised to assess causality in schizophrenia using Mendelian Randomisation [124, 125]. Mendelian Randomisation is underpinned by genomic variants derived from GWAS of an environmental exposure which explain a significant amount of variance such that they can be used as proxies for said exposure. Serum retinol levels have previously been subjected to GWAS, however, the relatively small sample (*N* ~ 5000) means that larger studies of the genetic determinants of retinol concentrations are needed to define appropriate instruments [126]. A limitation of this approach is that inference would be based on predicted serum retinol, which may not be the most ideal measure as evidence outlined in this review suggests that retinoic acid synthesis and signalling itself are likely the core pathology affecting this system in the disorder.
